# Non-Integrative Lentivirus Drives High-Frequency cre-Mediated Cassette Exchange in Human Cells

**DOI:** 10.1371/journal.pone.0019794

**Published:** 2011-05-23

**Authors:** Raul Torres, Aida García, Monica Payá, Juan C. Ramirez

**Affiliations:** Viral Vector Technical Unit, Fundación Centro Nacional de Investigaciones Cardiovasculares (CNIC), Madrid, Spain; Beckman Research Institute of the City of Hope, United States of America

## Abstract

Recombinase mediated cassette exchange (RMCE) is a two-step process leading to genetic modification in a specific genomic target sequence. The process involves insertion of a docking genetic cassette in the genome followed by DNA transfer of a second cassette flanked by compatible recombination signals and expression of the recombinase. Major technical drawbacks are cell viability upon transfection, toxicity of the enzyme, and the ability to target efficiently cell types of different origins. To overcome such drawbacks, we developed an RMCE assay that uses an integrase-deficient lentivirus (IDLV) vector in the second step combined with promoterless trapping of double selectable markers. Additionally, recombinase expression is self-limiting as a result of the exchangeable reaction, thus avoiding toxicity. Our approach provides proof-of-principle of a simple and novel strategy with expected wide applicability modelled on a human cell line with randomly integrated copies of a genetic landing pad. This strategy does not present foreseeable limitations for application to other cell systems modified by homologous recombination. Safety, efficiency, and simplicity are the major advantages of our system, which can be applied in low-to-medium throughput strategies for screening of cDNAs, non-coding RNAs during functional genomic studies, and drug screening.

## Introduction

Genetic modification of cells in human and experimental models is essential when deciphering gene-encoded information during developmental, physiological, and pathological states. A complete understanding of the mechanisms governing gene function is essential in areas such as development of regenerative and reparative medicine, gene therapy, genetic models of disease and reliable systems in drug discovery. One of the uses of homologous recombination (HR) is to generate stable “knock-in” cell lines (embryonic, iPSCs, or primary) with selectable markers expressing specific cDNAs or RNAi that make it possible to purify specific cell-derived cell types from a mixed population and to decipher their roles in cell stemness, differentiation, and fate, as well as in homeostasis and disease [1]. However, efficient and safe genetic targeting at stable and harmless loci remains elusive and is a limitation for low-to-medium throughput strategies. Genetic modification based on homologous recombination (HR), heterologous site-specific recombinase (SSR), and recombinase-mediated cassette exchange (RMCE) has been widely used in chromosomal targeting, although currently available technology is laborious [2], [3], [4], [5]. Zinc-finger nuclease–based and meganuclease-based [6], [7], [8] technologies have recently been designed for site-specific editing and are proving to be exceptionally promising tools, although they are time-consuming and expensive. In addition, these approaches are either inefficient or restricted in their applicability, thus preventing them from being used in large-scale functional genetic screening studies.

At present, the most efficient strategies for gene targeting combine tagged cellular systems containing recombination target sites (RTs) from phages (cre, phiC31)[9] or yeasts (flp)[10] with selected harmless genetic loci that are prone to recombination, for example, *ROSA*26 [11], *hprt* [12], [13], *AAVS*1 [6]. The generation of such tagged cells by HR is a relatively slow process that occurs at low frequency and requires selection and analysis of resistant clones. Tagged cells can be converted to express any sequence by gene replacement using genetic cassettes flanked by compatible RTs together with delivery of suitable recombinase. Gene targeting has been achieved in several loci of hES cells [11], [12] and site-specific recombinants can be enriched by RMCE at specific loci, with no apparent loss of the in vitro properties of hESCs [13].

No definite systems have been described to date for efficient cassette replacement by RMCE. Available approaches, such as application of cationic compounds, electroporation, and adenoviruses [14], [15], [16] , compromise the viability of cells and are complicated by restrictions that affect several cell types. In addition, overexpression of cre can be toxic for certain cells and tissues [17]. Consequently, novel approaches are necessary to improve RMCE frequencies and the limited expression of the recombinase. Vectors to ease cloning, manipulation, and high transduction efficiency in wide cell-types represent a challenge in hESC research [14]. Combined with tagged cells designed to promote efficient recombination and simple markers for the rescue of legitimate targeted clones, these vectors could make it possible to develop versatile cellular systems for gene studies.

We designed a novel strategy to meet most of the above requirements by combining the advantages of RMCE [2] with the powerful technology derived from integrase-deficient lentiviral vectors (IDLV). The strategy takes advantage of the easy management of lentivirus-derived vectors, the simple handling and cloning steps required for vector production, and the highly efficient transduction of almost every cell type assayed [18]. Envelope pseudotyping [19] and transductional targeting combining promoters and biosensors [20] have made it possible to transduce cells of several origins, tissue type, or developmental status using lentiviral vectors [21]. Lentiviral integration–dependent genotoxicity is no longer a major drawback, thanks to the generation of episomal viral circular forms by point-mutated integrase during IDLV replication [22]. These characteristics underlie the versatility of lentiviruses, irrespective of the target cell type (eg, primary or established cell lines, human or mouse embryonic stem cells).

Our strategy, which we call KAS*-*TRINA (see below), uses very high-frequency promoter trapping to detect and rescue cells undergoing legitimate gene replacement by recombination at a pre-targeted locus. We adopt a proof-of-principle approach using a novel strategy modeled on a human cell line, but which could be extended to other cell systems.

## Materials and Methods

### Cell culture

The human embryonic kidney cell lines HEK293A (ATCC #CRL-1573) and HEK293T (HEK 293FT, Invitrogen, Prat del Llobregat, Barcelona, Spain) were cultured under standard conditions in DMEM (Lonza, Lonza Ibérica SA, Barcelona, Spain) supplemented with 1% Glutamax (Invitrogen), 10 mg/ml antibiotics (penicillin streptomycin) and 10% foetal bovine serum (Gibco, Invitrogen).

After the selection of cells with hygromycin B solution (Sigma Aldrich) at 50 µg/mL, resistant colonies were stained with cristal-violet on methanol fixed plates, washed and air dried.

### Plasmid construction

To construct the hROSA26-targeting vector, we used the homology arms previously described by Keller et al. [11]. We introduced the homology arms amplified by PCR using the RP11-58B17 BAC clone as a template (BACPAC Resource Center (BPRC), Children's Hospital Oakland Research Institute, California, USA) into the pUC57 empty backbone, named pK0.

pKAS (from *K*nock-down/in *A*cceptor *S*ite) was cloned using a building block synthesized (GenScript, GenScript USA Inc. Piscataway, NJ, USA) named pK1 containing the lox2272 site, strong stop sequence and, loxP site and the hygromycin B phosphotransferase (hph) gene. The PGK promoter obtained from the pLVX.CMV.AcGFP.PGK.Puro vector (Clontech, Saint-Germain-en-Laye, France) was cloned in the NotI and PstI sites in pK1. In the following step, we introduced the neo cassette from pcDNA 3.1 (Invitrogen) flanked by XmnI–SalI sites. Finally, we cloned the whole cassette into pK0 (containing the the hROSA26 homology arms) using the AscI–MluI restriction sites.

The plasmid used to produce the RMCE event (pTRINA, from *Tr*ansfer *In*to *A*cceptor) was synthesized by GenScript. We amplified this recombineering cassette using specific primers and the product was cloned in the reverse orientation of the pRRLsin18, a third generation lentiviral shuttle plasmid [23], [24] to generate pLV.TRINA.

pMDLg/pD64VRRE plasmid was generated by PCR mutagenesis of the nucleotides encoding the aspartic acid substituted by valine (D64V) using pMDLg/pRRE plasmid as template.

pRRLLsin18.CMVCre was cloned by ligation of the cre cDNA obtained from LV-Cre-SD (12105, Addgene, Cambridge, MA, USA) in the XbaI-SalI sites of donor pRRLsin18CMV lentiviral shuttle vector.

All constructs were verified by sequencing with an ABIPrism 3000 sequencer (Applied Biosystems, Invitrogen).

### DNA transfection

Cells were transfected with endotoxin-free DNA (Qiagen, Las Matas, Madrid, Spain) and transfections were carried out in 6-well plates unless otherwise stated. HEK293T and HEK293A cells were transfected by the calcium-phosphate method using 5 µg of pKAS targeting plasmid and an eGFP control plasmid (pEGFP-N1, Clontech) linearized using *MluI* and *ScaI* restriction enzymes (New England Biolabs, IZASA, S.A. Barcelona, Spain), respectively. After transfection cells were plated in their appropriate medium in 6-cm tissue culture dishes (Nunc, LabClinics S.A. Barcelona, Spain). Forty-eight hours later, G418 (Invitrogen) was added to the culture medium in a final concentration of 500 µg/mL. Medium was changed every 2–3 days, until isolated clones were visible (10–14 days).

### Quantification of transgene copy numbers by qPCR analysis

The genomic DNA was extracted following standard procedure and serial dilutions quantitated using NanoDrop ND 1000 Spectrophotometer (NanoDrop Technologies, Bonsai Tecnologies Group SL, Alcobendas, Madrid, Spain). Real-time PCR was performed using the SYBR Green methodology. The number of integrated copies of pKAS plasmid in the 293AKAS cells was measured as described in Butler et al. [25] with modifications. PCR efficiency was examined with five dilutions of genomic DNA and the specificity of individual gene primers was validated by the melting curve at the end of each PCR assay. Standard curves were obtained with diluted amounts of the pKAS plasmid from 0.01 pg to 100 ng, corresponding 10^2^–10^9^ copies. Ct values obtained upon amplification of neomicin, PGK and hygromycin regions contained in pKAS using the specific primers listed below were interpolated and the relative number of copies calculated. Cell equivalents were calculated using Ct values of the single copy gene Gusb on diluted samples of the genomic DNA. The qPCR reaction was carried out under the following conditions: template denaturation at 95°C for 10 min, followed by 40 cycles of denaturation at 95°C for 15 s, annealing at 60°C for 20 s, and extension at 72°C for 30 s. The primers used were:

qPGK Fw: CTTTGCTCCTTCGCTTTCTG

qPGK Rv: CGGAGATGAGGAAGAGGAGA

Product: 171 bp

qNEO Fw: TGAATGAACTGCAGGACGAG

qNEO Rv: ATACTTTCTCGGCAGGAGCA

Product: 171 bp

qHYGRO Fw: CTCGATGAGCTGATGCTTTG

qHYGRO Rv: GATGTTGGCGACCTCGTATT

Product: 165 bp

Gusb Forward: GTAGGGACAAGAACCACCCC

Gusb Reverse: TTGCTCACAAAGGTCACAGG.

Product: 148 bp

Standard PCR was performed using the following conditions: template denaturation at 95°C for 1 min, followed by 30 cycles of denaturation at 94°C for 30 s, annealing at 61°C for 30 s, and extension at 72°C for 1.5 min, and a final extension of 5 min.

The primers used were: for pKAS integration detection

Fw primer #1 (Neo) GAAATGACCGACCAAGC

Rv primer #2 (R26LA) GCCCCTCAAATCTTACAGC

Product: 1450 bp

and for RMCE product detection

Fw primer #3 (R26SA) GCCGAGACTTCTGGATG

Rv primer #4 (Cherry) GAACTCCTTGATGATGGCC

Product: 1250 bp.

### Lentiviral vector production and transduction

Third-generation self-inactivating VSV-G-pseudotyped lentiviral vectors were produced by transient transfection into HEK293T cells as previously described [26]. Briefly, cells were seeded at 1.1×10^7^ cells/dish in 15-cm dishes the day before transfection. Cells were transfected by the calcium phosphate method with 3 µg pRSV-Rev, 3.75 µg pMD.2G (VSV-G), 13 µg pMDLg/pRRE (or 13 µg pMDLg/pD64VRRE for production of non integrative lentiviral vectors) and 35 µg of transfer plasmid (pTRINA, pRRL18sin.CMV-Cre or pRRLsin18.CMV-eGFP, unpublished results). The medium was collected after 48 h, cleared by low-speed centrifugation, and filtered through 0.45-µm-pore-size PVDF filters. Viral stocks were concentrated by ultracentrifugation in SW28 Beckman rotor at 90,000 g (26,000 rpm) for 2 h at 4 °C. Pellets containing lentivirus were air dried and resuspended O/N at 4°C in 400–600 µl of media. Viral titres were calculated by FACS analysis on transduced HEK293T cells when vectors expressed fluorescent proteins (transduction units/ml) and particles quantitated by qPCR on supernatants (particles/ml). Values were around 10∧8 to 10∧9 TU/ml and in a 1∶100 TU/particles ratio. Cells were transduced at different multiplicity of infection (MOI) in media containing polybrene (8 µg/ml, final concentration). After 6 hours inocula was replaced with fresh medium, and 72 hours later the cells were analyzed by FACS or by starting hygro selection (50 µg/mL to select HEK293A).

### Western blot

Proteins were extracted following standard procedures in the presence of Complete Protease Inhibitor Cocktail Tablets (Roche Applied Science). Western blots were carried out by standard methods on proteins transferred to PVDF using TransFi (Invitrogen). Membranes were probed for cre with monoclonal anti-Cre antibody (Novagen, EMD, Merck – España, Madrid Spain) (1/2000) or anti-alpha tubulin (AbCam, Cambridge, UK) (1/5000) in PBS/0.1% Tween-20 (PBSTween). Secondary antibodies were HRP-conjugated goat antimouse IgG (Santa Cruz Biotechnology, Inc., Heidelberg, Germany), and blots were developed with ECL (GE Healthcare, Alcobendas, Spain).

### Cytometry analyses

Flow cytometry analysis was performed after 72 hours post-transduction. Cells were trypsinized and collected, washed twice in culture-grade 1X PBS and analyzed in a FACS DIVA (Becton Dickinson, San Agustín del Guadalix, Madrid, Spain) sorter with an appropriate laser for CherryFP excitation. In every case, 10,000 events were counted in triplicate.

## Results

### Design and strategy

**Figure 1:**
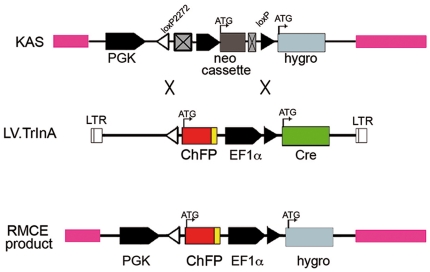
Cre recombinase-mediated insertion and cassette exchange strategy. Structure of the genetic landing pad (KAS) and the incoming construct (LV.TRINA) showing the more relevant elements: promoters PGK, EF-1α and SV40 in the neo cassette (black arrows), reporter genes (coloured boxes), recombinase cre (green box), translation starting point (bended arrows), and polyA signals (crossed boxes). Triangles represent the loxP (black) and lox2272 (white) sites. ROSA26 homology arms are pink-colored and lentiviral LTRs drawn as clear boxes flanking the KAS and TRINA cassette respectively. For simplicity the CHYSEL and multicloning site downstream of the neo cassette are not indicated. The expected resulting endpoints after RMCE reaction are depicted below. Symbols are kept for simplicity. ChFP, cherry fluorescent protein; LTR, long terminal repeat.

The system was designed to promote RMCE on pre-tagged cells with a genetic docking site containing heterospecific (incompatible) RT sites (loxP and lox2272) [27], [28] flanking a promoter-trapped reporter gene cassette and targeted by the second cassette delivered by IDLV concomitantly expressing cre recombinase in a self-limiting manner (Fig. 1). Heterospecific RT sites are less susceptible to intra-molecular excision/inversion. Lox2272 contains two mutations in the 8 bp core compare to wt loxP site and intra-molecular reaction is lower than 0,5% compared to directional inter-molecular exchange ([29]).

To test our approach, we constructed a targeting vector (Fig. 1) containing a selectable cassette (pKAS, from *K*nock-down/in *A*cceptor *S*ite) flanked by two homologous arms of the human *ROSA*26 locus [11]. The cassette contains the weak but ubiquitously functional PGK promoter [30] followed by strong stop signal and flanked by loxP and lox2272 an expression cassette of the gene *neo*, which encodes neomycin resistance controlled by the early promoter and polyA signal from SV40. This cassette is followed by a promoterless reporter–selection cassette containing the *hph* (hygromycin B phosphotransferase) gene, which encodes hygromycin resistance. Cells bearing this genetic landing pad are neo^R^ selected and undergo RMCE. The lentivirus shuttle plasmid backbone (pTRINA, from *Tr*ansfer *In*to *A*cceptor) contains the promoterless gene of interest (GOI)–reporter cassette flanked by the same heterospecific lox sites. The latter consists of a fluorescent marker (cherry fluorescent protein [ChFP]) fused to the Picornaviridae porcine teschovirus CHYSEL sequence 2A [31] and a multicloning site to clone every GOI. Finally the EF1alpha promoter drives the expression of cre coding cassette placed downstream of the loxP site. The complete construct was cloned in an inverted position relative to viral LTR in order to preclude premature transcriptional termination by polyA signals present in the cassette that might prevent generation of genomic mRNAs during production of lentiviruses.

Selected cells containing the genetic docking site are transduced by VSV-pseudotyped IDLV particles and, upon reverse transcription, nuclear lentiviral DNA structures of genome unit length [25] are transcriptionally active to express cre recombinase from the constitutive EF1alpha promoter. RMCE is initiated by exchanging the cassette contained in the recombinant lentiviral genome flanked by heterospecific loxP sites with that present in the genetic docking site. The promoter trap design allows for easy isolation and characterization of the targeted cells by double marker rescue (antibiotic resistance and fluorescent protein). RMCE can be performed with heterospecific target sites that are equally or inversely oriented; we have selected the latter version, anticipating that unwanted intersite-interactions would lead, in this case, to inversion and not to excision preventing the expression of *hph* gene from the PGK promoter.

### Episomal recombination using an engineered genetic landing pad

**Figure 2:**
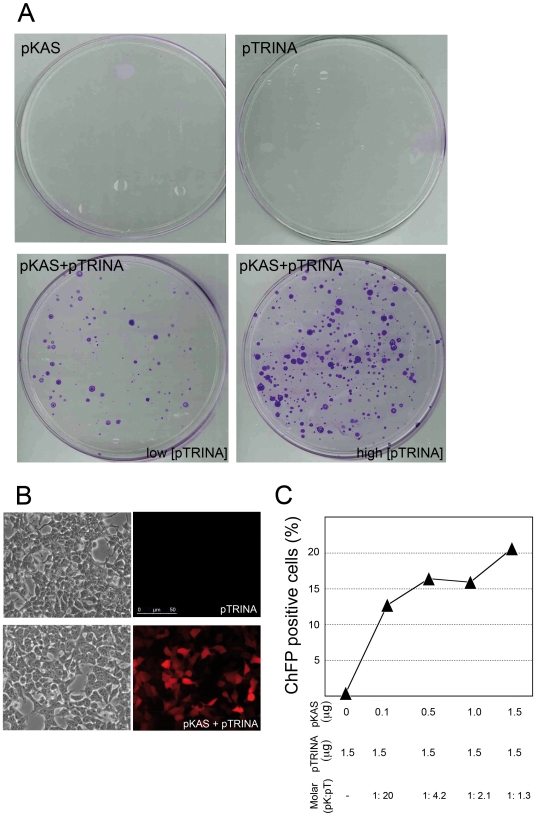
Plasmid-mediated RMCE by transient transfection. (A) Hygromycin-resistant colonies obtained upon transfection of HEK293A cells with the indicated plasmids containing the genetic landing pad (pKAS) or the transfer plasmid (pTRINA) expressing cre-recombinase at two different relative amounts (low and high [pTRINA]). Colonies were stained after ten days under conditions of selection. (B) Microphotographs under brightfields (left) and fluorescent light (right) of the transfected cultures with the indicated plasmids. (C) Number of cells expressing cherry fluorescent protein (ChFP+) upon RMCE on HEK293A cells measured by FACS analysis. Variable amounts of pKAS were used with a constant amount of pTRINA as is indicated. Transfections were made in duplicate, and the results shown in both A and B are from one representative experiment out of three.

We tested the feasibility of our strategy in human HEK293 cells by first addressing the ability of the cre expression plasmid, pTRINA, to catalyze RMCE between two episomal substrates. We used an assay based on transient co-transfection of supercoiled pKAS and a pTRINA and measured both the frequency of hygromycin-resistant (hygro^R^) cell colonies and the percentage of cells expressing of ChFP. Neither hygro^R^ colonies (Fig. 2A) or ChFP+ cells (Fig. 2B at zero value on the horizontal axis, Fig. 2C) appeared when cells were transfected with either pKAS or pTRINA alone. On the contrary, hygro^R^ colonies (between 75–300 colonies/assay; Fig. 2A) and cherry-expressing cells (ChFP+) (between 10–20%; Fig. 2B, 2C) were obtained only when both plasmids were transfected together. Considering transfection efficiency close to 95% (data not shown), the frequency of hygro^R^ or ChFP+ generated when only one plasmid was transfected is lower than 3×10^−6^. Remarkably the frequencies of RMCE between two episomal substrates ranging from 3.5×10^−4^ (ChFP+) to 1.5×10^−5^ (hygro^R^), values that are indicative of a suitable cassette exchange in our system and that are similar to data for RMCE in selected tagged cells [13]. Discrepancies in the frequencies obtained for hygro^R^ or ChFP+ could stem from relative expression levels of the two reporter genes to select a positive cell, as levels required for hygromycin resistance are not comparable with the greatly enhanced sensitivity required to detect a fluorescent single cell by FACS analysis ([32]).

We also used flow cytometry to quantify the number of cells expressing ChFP+ cells 72 hours post-transfection under variable relative amounts of pKAS together with fixed amounts of pTRINA. Fig. 2C shows that with a 15-fold molar increase in the relative amount of pKAS carrying the genetic landing pad (1∶20 compared to 1∶1.3 ratio), the total number of ChFP+ cells increases nearly 2-fold, suggesting that the availability of the docking site is a limiting element for RMCE under conditions of sufficient levels of transient cre expression. When the reversible assay was carried out, we found that up to10-fold lower molar ratio of pTRINA related to pKAS is efficient enough to promote RMCE (data not shown).

Together the data obtained using either hygro (Fig. 2A) or ChFP (Fig. 2B, 2C) markers, demonstrate that plasmids bearing the genetic docking and the cre expressing cassettes are both required to generate promoter trapping. Remarkably, the number of RMCE events can vary with the relative amount of the cre-expressing plasmid (Fig. 2A).

We hypothesized two different scenarios for the generation of ChFP+ and hygro^R^ colonies: (i) random integration of pTRINA and of pKAS accompanied by independent promoter trap events; and (ii) legitimate cassette exchange, when recombination occurs simultaneously between two pairs of heterospecific RT by the action of cre. Given that the spontaneous frequency of appearance of ChFP+ and hygro^R^ cells is lower than 3×10^−6^, the use of a double checkpoint renders scenario 1 unlikely, as the frequency of appearance of double-positive cells by two independent random promoter trap events will be close to one cell in one billion of the transfected cells. The enrichment strategy allows direct selection of SSR, as random integration is likely to be transcriptionally silent and will not resist hygromycin selection or become red fluorescent.

### Integrase-deficient lentiviruses can promote RMCE in randomly integrated copies of genetic docking target

**Figure 3:**
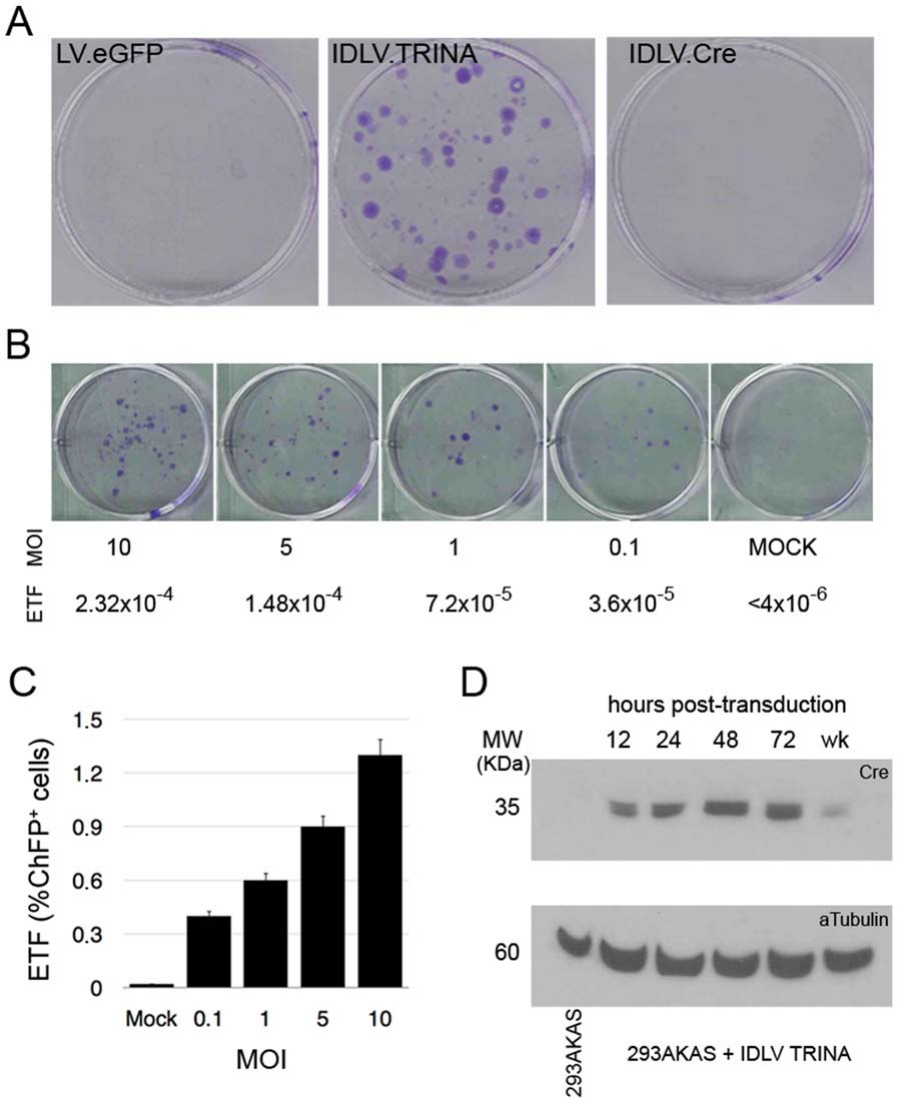
Randomly chromosomal integrated copies of the genetic landing pad KAS are targeted by IDLV expressing the TRINA cassette. HEK293A cells resistant to G418 (293AKAS) were pooled and transduced with low MOI (2 transduction unit/cell) of IDLV-TRINA. (A) Transduced cells were selected with hygromycin and the colonies counted. The figure shows stained plates after selection of cells MOCK-transduced (LV.eGFP, left) or transduced with IDLV-TRINA (center) or IDLV-Cre (right). (B) Quantification of RMCE frequency in 293AKAS cells by culturing under selection conditions with hygromycin upon transduction at different MOI with IDLV-TRINA . (C) Quantification of RMCE frequency in 293AKAS cells by FACS analysis of ChFP+ upon transduction at different MOI with IDLV-TRINA as in B. Triplicate cultures were transduced, FACS analyzed in triplicate. Average +/−SD is represented. (D) Western blot to detect cre expression in pooled 293AKAS cells at different points after transduction with IDLV.TRINA (MOI 2 TU/cell). The alpha-tubulin protein was used as a loading control (bottom panel). Results in A and C are from one representative experiment out of three. ETF: Effective Targeting Frecquency: ratio between hygro^R^ (B) or ChFP+ (C) and the number of total G418^R^ cells plated for transduction.

We showed that pTRINA catalyses site-specific cassette exchange between two episomal plasmids flanked by heterospecific lox sites. We next investigated whether RMCE can also be achieved between a chromosomal DNA substrate and circular intermediate lentiviral genomes formed during reverse transcription in the absence of a functional integrase. As a first approach, we targeted randomly integrated genetic landing pad copies on pooled (n>80) neo^R^ HEK293A cells (293AKAS) obtained by transfection with pKAS and G418 selection. Transduction at low MOI (2 transduction units/cell) of the lentivirus IDLV-TRINA into the pooled 293AKAS was applied to measure the frequency of RMCE by the lentivirus. Upon cre recombinase-catalyzed SSR, the promoterless hygromycin and ChFP genes are placed under the transcriptional control of PGK and EF1alpha promoters, respectively. Both hygro^R^ and red colonies (ChFP-expressing cells) were counted as an approximate quantification of the RMCE process. As shown in Fig. 3A, only when cre recombinase is expressed from the IDLV virus (IDLV-TRINA), 293AKAS cells were able to become ChFP+ or hygro^R^ cells. Remarkably, all hygro^R^ phenotypes also displayed the ChFP+ phenotype, as observed by fluorescence microscopy analysis, thus demonstrating that legitimate RMCE was occurring (data not shown). A rough estimation of the frequencies at this MOI is, on average, 400 hygro^R^ colonies in 1×10^6^ neo^R^ cells, close to an RMCE frequency of 4×10^−4^, or close to1×10^−2^ in the case of ChFP+ measurement.

The directionality in the recombination reaction between loxP and lox2272 sites explains the performance revealed by the frequency of hygro^R^ cells as well as on the appearance of cherry-expressing cells. Although cre-mediated recombination between incompatible lox sites is almost negligible [33], the rescue of hygro^R^ cells could be due either to the excision reaction in cis in the pKAS construct leading to the *hph* gene joined downstream to the PGK promoter, or to leaky expression resulting form RNA 3′readthrough [34]. To study these phenomena, we transduced 293AKAS cells at low MOI (2 TU/cell) with a lentivirus expressing cre recombinase alone (pRRLsin18.CMVcre) and counted the number of hygro^R^ colonies obtained. As shown in Fig. 3 (right), not a single colony was obtained, thus representing a frequency below 1×10^−6^ illegitimate recombination events in our system. Such values cannot be raised by increasing the MOI, given that up to 50 TU/cell were used and did not yield hygro^R^ colonies (data not shown). These data strongly support the idea that our strategy enables the direct selection of RMCE, because random integration is transcriptionally silent and cells do not survive G418 or hygromycin selection.

### Viral transduction titrates the efficiency of RMCE

Having shown that the non-integrative lentivirus can considerably increase the frequencies of RMCE, we performed assays to assess whether the strategy could be improved merely by modifying the lentivirus transduction procedure. As HEK293A cells are easily transduced, we studied the effect of the MOI on the frequency of SSR. Fig. 3 shows that by increasing the amounts of IDLV.TRINA, the number of hygro^R^ colonies (B) and ChFP+ cells (C) parallels the MOI in the range of 0.1 to 10 TU/cell. The relative effective targeting frequency (ETF), measured as the ratio between number of hygro^R^/ChFP+ cells and total number of G418^R^ 293AKAS cells plated, is the most reliable measurement of RMCE, given that it is difficult to detect the total number of cells transduce as there is no an additional reporter gene. Then there is no experimental data to calculate the absolute targeting frequency as the proportion of promoter trapped cells related to the number of transduced cells. ETF is between 2×10^−4^ and 7×10^−5^ hygro^R^ and, importantly, between 1×10^−2^ and 8×10^−3^ ChFP+ at MOIs of 10 TU/cell and 5 TU/cell. These values fell 10- to 20-fold when low MOIs were used. The frequencies obtained at higher MOI are 10-fold higher than published for lipofection and 300-fold higher than for electroporation by antibiotic resistance [13]. However, if ChFP+ values are considered, then 1,000- to 10,000-fold increases were obtained. The result suggests that the level of cre, which is controlled by its own activity as the expression cassette is flanked by heterospecific loxP sites (Fig.1) and diluted with cell division, is the major bottleneck in the RMCE. We have addressed the kinetics of expression of cre recombinase at different time points upon transduction of 293AKAS cells with IDLV.TRINA at MOI of 2 TU/cell. As shown in Fig. 3D, levels of cre increases from 12 to 72 hours post transduction and start decreasing to almost undetectable levels at seven days post transduction. Similar results were observed by immunfluorescence on transduced cells, as at 5 days post transduction the protein is almost undetectable (data not shown). Besides, the lack of cre recombinase expression in untransduced cells supports the idea that expression of cre encoded by IDLV.TRINA transduction is necessary to obtain the ChFP+ and hygro^R^ phenotypes.

However, importantly, numbers of cells undergoing RMCE could easily be raised with a concomitant increase in the MOI, a situation resembling that of cells which are difficult to transduce, whereas at MOIs low enough to ensure minimal—if any—effect on cellular viability or integrity (2 TU/cell), efficiency is high (1×10^−3^).

We also studied the stability of the expression of both markers. Cultures of hygro^R^ cells were maintained under selective conditions for four weeks and periodically analyzed using FACS to quantitate the absolute number of ChFP+ cells related to hygro^R^ . Although numbers of ChFP+ cells were slightly different, nearly 60% were positive after one month in culture (data not shown). Even when cells undergo RMCE correctly, i.e. by positioning each genetic element in the predicted manner (thus allowing the selection of the targeted cells), expression for the fluorescent marker was not stable under hygromycin selection, a phenomenon described in other RMCE strategies [13].

### IDLV-TRINA targets genomic landing pad by legitimate RMCE

**Figure 4:**
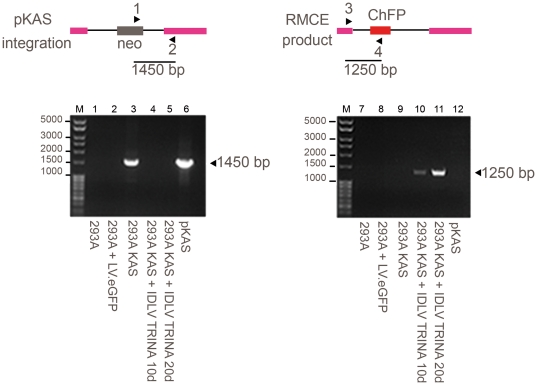
PCR analysis of genomic DNA isolated to verify RMCE reaction. PCR analysis of genomic DNA extracted from the HEK293A cells (lane 1 and 7), or transfected with pKAS (293AKAS) (lanes 2 to 5 and 8 to 11). Cells were transduced with control virus IDLV.CMVeGFP (lanes 2 and 8), IDLV.TRINA (lanes 4, 5, 10 and 11), or non-transduced (lanes 3 and 9). Positive control is DNA from pKAS (lanes 6 and 12). The 1250 bp band is indicative of RMCE between KAS and TRINA cassettes and the 1450 bp corresponds to integrated pKAS band. IDLV.TRINA/10 and IDLV.TRINA/20 correspond to DNA obtained after from cultures selected during10 and 20 days with hygromycin respectively. The sizes of the DNA fragments in the marker line M are indicated.

We verified the recombination reaction by PCR. Genomic DNA was prepared from neo^R^ cultures of 293AKAS selected with hygromycin for 10 and 20 days after transduction with IDLV.TRINA (MOI 2 transduction units/cell). Control DNA was obtained from untransduced HEK293A and 293AKAS cultures transduced with an unrelated-IDLV expressing the eGFP reporter gene (pRRLsin18.CMVeGFP). Conventional PCR was conducted using specific primers to detect integrated KAS or the RMCE product (Fig. 4) that generated bands of 1450 bp and 1250 bp, respectively. We also studied the random integration of the IDLV.TRINA on the same samples by qPCR using LTR-specific primers [25] and found less than one integration copy/cell according to standardized curves (data not shown). The predicted 1250 bp product indicating cassette exchange between KAS and TRINA only appeared when cells were transduced with IDLV.TRINA (Fig. 4 lane 10 and 11). In contrast, no PCR product was generated with DNA from control cells (HEK293A, 293AKAS and 293AKAS.IDLVeGFP; Fig. 4 lanes 7, 8 and 9). Importantly, the absence of the 1.5 Kb band in IDLV.TRINA transduced 293AKAS cultures (Fig. 4, lanes 4 and 5) strongly supports the idea that the RMCE reaction in our conditions is highly efficient. Then all the copies of pKAs randomly integrated that were stimated in 1,8 copies per cell by qPCR, are submitted to RMCE.

## Discussion

The purpose of the experiments presented here was to provide proof-of-principle that non-integrative lentiviruses can be used in cell-mediated strategies for freely modifying the murine and human genome by RMCE. We showed that legitimate site-specific recombination governed by transcription from an integration-deficient lentiviral vector (IDLV.TRINA) promotes exchange of replicative viral intermediates flanked by heterospecific lox sites with equally flanked chromosomal DNA substrate (KAS). The system is simple, yet highly efficient, and promotes RMCE at frequencies not previously reported, to our knowledge, in selected cells harboring integrated docking sites, although we targeted randomly integrated copies instead of using HR. The activation of the reporters (ChFP and *hph*) relies on the autolimited expression of the recombinase and is not based on the cellular repair machinery or cre-like activity present.

Despite restrictions due to the specific features of the strategy, we observed that 100% of the recovered clones were selected as a result of RMCE by coupling promoter trap strategy with SSR as published elsewhere [2], [13], [16], [27], [35]. However, it is noteworthy that frequencies between 1×10^−3^ and 1×10^−2^ are 1,000-fold to 100,000-fold higher than previously reported using either lipofection or electroporation, respectively. Besides highlighting the beneficial features raised from properties of lentiviral vectors, i.e. easy cloning and production of the vector and high level of cell viability upon transduction, we showed that the major advantages of KAS/TRINA are based on the following: (i) self-limiting expression of the cre recombinase delivered by the viral vector; (ii) transient expression from the IDLV; and, finally (iii) scalability of the frequency of RMCE simply by increasing the MOI for transduction. Importantly, frequencies of RMCE are two log units above the very low frequency of integration of the IDLV, estimated between 1×10^−4^ to 2×10^−5^ integrants/viral genome copies [36], [37], which renders its selection during the procedure negligible, given frequencies of RMCE close to 1% of the transduced cells at low MOIs (Fig. 3C). We observed that our results were proof-of-principle by using cells prone to transduction and randomly integrated copies. The number of integrations of the docking cassette in the pooled 293AKAS cells is around 1.8 copies per cell, a data that allows to compare the frequencies obtained using our strategy with those obtained with RMCE on tagged cells at single specific loci [12], [16], [38], then targeting 2 copies per cell. Indeed, given that pKAS is inserted in multiple loci, positional effects due to random insertions underestimate our frequencies of RMCE compared to gene replacement at *hprt* or *ROSA*26 loci that are prone to recombination, and RMCE appears to address all KAS-targets (PCR test in Fig. 4). Limited expression of cre recombinase is controlled by its own enzyme activity, as it expression cassette in pTRINA is flanked by lox sites and the non-episomal nature of the IDLV intermediates switches the expression off.

Our genetic approach, Kas/TRINA, could be used to modify refractory cells [14] by increasing the amount of virus or establishing efficient and selective transduction protocols. This system is affordable, efficient, and easy, and can be applied in large-scale assays or low-to-medium throughput assays. Therefore, we consider that our strategy may be a useful tool for genetic manipulation, not only of tagged cell lines, but also of ESCs and iPSCs. Although similar to the strategies used in other reports [2], our drug/colored selectable system is a predictable and highly reproducible platform to study gene function in almost any cell type. However, the novelty of delivering recombinase in the same lentiviral vector in a non-integrative and self-limiting manner by means of lox flanking eliminates enzyme toxicity [17] and the need for additional and independent gene transfer [39].

The availability of cell lines tagged at predefined loci (*ROSA*26, *AAVS*1, *hprt*) with heterospecific lox sites in combination with att or frt sequences should facilitate—through repeated transduction with IDLV encoding different recombinases—the study of multiple genetic elements. The strategy can be further developed using engineered transgenes containing specific recombinase sites and will enable the generation of secondary cells lacking a particular factor and leaving only a localized genetic scar. Our strategy can be applied in the screening of early molecular events leading to stem cell differentiation and disease, in genetic simulation of disease models, and, importantly, in genetic studies using iPSC technology. In addition, the genetic homogeneity of the cells makes chemical and genetic screening approaches feasible, easy, comparable, and reliable. The efficiency and lengthy process of generating knock-in mouse models should be shortened by combining established ES cell lines bearing the docking KAS or KAS-like derivatives with available lentiviral technology for microinjection to generate KI or KD by RMCE.

We further demonstrate the powerful technology of viral vectors based on non-integrative lentivirus, an achievement that will contribute to the development of novel and safer strategies for gene transfer.

## References

[1] Branda CS, Dymecki SM (2004). Talking about a revolution: The impact of site-specific recombinases on genetic analyses in mice.. Dev Cell.

[2] Wirth D, Gama-Norton L, Riemer P, Sandhu U, Schucht R (2007). Road to precision: recombinase-based targeting technologies for genome engineering.. Curr Opin Biotechnol.

[3] Grindley ND, Whiteson KL, Rice PA (2006). Mechanisms of site-specific recombination.. Annu Rev Biochem.

[4] Lewandoski M (2001). Conditional control of gene expression in the mouse.. Nat Rev Genet.

[5] Song H, Chung SK, Xu Y (2010). Modeling disease in human ESCs using an efficient BAC-based homologous recombination system.. Cell Stem Cell.

[6] Hockemeyer D, Soldner F, Beard C, Gao Q, Mitalipova M (2009). Efficient targeting of expressed and silent genes in human ESCs and iPSCs using zinc-finger nucleases.. Nat Biotechnol.

[7] Redondo P, Prieto J, Munoz IG, Alibes A, Stricher F (2008). Molecular basis of xeroderma pigmentosum group C DNA recognition by engineered meganucleases.. Nature.

[8] Lombardo A, Genovese P, Beausejour CM, Colleoni S, Lee YL (2007). Gene editing in human stem cells using zinc finger nucleases and integrase-defective lentiviral vector delivery.. Nat Biotechnol.

[9] Groth AC, Calos MP (2004). Phage integrases: biology and applications.. J Mol Biol.

[10] Birling MC, Gofflot F, Warot X (2009). Site-specific recombinases for manipulation of the mouse genome.. Methods Mol Biol.

[11] Irion S, Luche H, Gadue P, Fehling HJ, Kennedy M (2007). Identification and targeting of the ROSA26 locus in human embryonic stem cells.. Nat Biotechnol.

[12] Zwaka TP, Thomson JA (2003). Homologous recombination in human embryonic stem cells.. Nat Biotechnol.

[13] Di Domenico AI, Christodoulou I, Pells SC, McWhir J, Thomson AJ (2008). Sequential genetic modification of the hprt locus in human ESCs combining gene targeting and recombinase-mediated cassette exchange.. Cloning Stem Cells.

[14] Braam SR, Denning C, van den Brink S, Kats P, Hochstenbach R (2008). Improved genetic manipulation of human embryonic stem cells.. Nat Methods.

[15] Eiges R, Schuldiner M, Drukker M, Yanuka O, Itskovitz-Eldor J (2001). Establishment of human embryonic stem cell-transfected clones carrying a marker for undifferentiated cells.. Curr Biol.

[16] Sorrell DA, Robinson CJ, Smith JA, Kolb AF (2010). Recombinase mediated cassette exchange into genomic targets using an adenovirus vector.. Nucleic Acids Res.

[17] Silver DP, Livingston DM (2001). Self-excising retroviral vectors encoding the Cre recombinase overcome Cre-mediated cellular toxicity.. Mol Cell.

[18] Naldini L, Verma IM (2000). Lentiviral vectors.. Adv Virus Res.

[19] Cronin J, Zhang XY, Reiser J (2005). Altering the tropism of lentiviral vectors through pseudotyping.. Curr Gene Ther.

[20] Brown BD, Gentner B, Cantore A, Colleoni S, Amendola M (2007). Endogenous microRNA can be broadly exploited to regulate transgene expression according to tissue, lineage and differentiation state.. Nat Biotechnol.

[21] Delenda C (2004). Lentiviral vectors: optimization of packaging, transduction and gene expression.. J Gene Med.

[22] Banasik MB, McCray PB (2010). Integrase-defective lentiviral vectors: progress and applications.. Gene Ther.

[23] Zufferey R, Dull T, Mandel RJ, Bukovsky A, Quiroz D (1998). Self-inactivating lentivirus vector for safe and efficient in vivo gene delivery.. J Virol.

[24] Dull T, Zufferey R, Kelly M, Mandel RJ, Nguyen M (1998). A third-generation lentivirus vector with a conditional packaging system.. J Virol.

[25] Butler SL, Hansen MS, Bushman FD (2001). A quantitative assay for HIV DNA integration in vivo.. Nat Med.

[26] Paya M, Segovia JC, Santiago B, Galindo M, del Rio P (2006). Optimising stable retroviral transduction of primary human synovial fibroblasts.. J Virol Methods.

[27] Araki K, Araki M, Yamamura K (1997). Targeted integration of DNA using mutant lox sites in embryonic stem cells.. Nucleic Acids Res.

[28] Lee G, Saito I (1998). Role of nucleotide sequences of loxP spacer region in Cre-mediated recombination.. Gene.

[29] Kolb AF (2001). Selection-marker-free modification of the murine beta-casein gene using a lox2272 [correction of lox2722] site.. Anal Biochem.

[30] Ramezani A, Hawley TS, Hawley RG (2000). Lentiviral vectors for enhanced gene expression in human hematopoietic cells.. Mol Ther.

[31] de Felipe P (2004). Skipping the co-expression problem: the new 2A “CHYSEL” technology.. Genet Vaccines Ther.

[32] Rodolosse A, Barbat A, Chantret I, Lacasa M, Brot-Laroche E (1998). Selecting agent hygromycin B alters expression of glucose-regulated genes in transfected Caco-2 cells.. Am J Physiol.

[33] Siegel RW, Jain R, Bradbury A (2001). Using an in vivo phagemid system to identify non-compatible loxP sequences.. FEBS Lett.

[34] Zaiss AK, Son S, Chang LJ (2002). RNA 3′ readthrough of oncoretrovirus and lentivirus: implications for vector safety and efficacy.. J Virol.

[35] Qiao J, Oumard A, Wegloehner W, Bode J (2009). Novel tag-and-exchange (RMCE) strategies generate master cell clones with predictable and stable transgene expression properties.. J Mol Biol.

[36] Qasim W, Vink CA, Thrasher AJ (2010). Hybrid lentiviral vectors.. Mol Ther.

[37] Yanez-Munoz RJ, Balaggan KS, MacNeil A, Howe SJ, Schmidt M (2006). Effective gene therapy with nonintegrating lentiviral vectors.. Nat Med.

[38] Sakurai K, Shimoji M, Tahimic CG, Aiba K, Kawase E (2010). Efficient integration of transgenes into a defined locus in human embryonic stem cells.. Nucleic Acids Res.

[39] Nolden L, Edenhofer F, Haupt S, Koch P, Wunderlich FT (2006). Site-specific recombination in human embryonic stem cells induced by cell-permeant Cre recombinase.. Nat Methods.

